# A NIR-Light-Driven Twisted and Coiled Polymer Actuator with a PEDOT-Tos/Nylon-6 Composite for Durable and Remotely Controllable Artificial Muscle

**DOI:** 10.3390/polym14030432

**Published:** 2022-01-21

**Authors:** Inwook Hwang, Seongcheol Mun, Hyungcheol Shin, Sungryul Yun

**Affiliations:** Human Enhancement & Assistive Technology Research Section, Electronics and Telecommunications Research Institute (ETRI), Gajung-ro, Daejeon 218, Korea; inux@etri.re.kr (I.H.); scmun@etri.re.kr (S.M.); shin@etri.re.kr (H.S.)

**Keywords:** photo-thermal, contractile strain, TCN, NIR

## Abstract

In this paper, we proposed a novel light-driven polymer actuator that could produce remotely controllable tensile stroke in response to near infrared (NIR) light. The light-driven polymer actuator was composed of a twisted and coiled nylon-6 fiber (TCN) and a thin poly(3,4-ethylenedioxythiophene) doped with *p*-toluenesulfonate (PEDOT-Tos) layer. By adopting dip-coating methodology with thermal polymerization process, we constructed a thin and uniform PEDOT-Tos layer on the surface of the three-dimensional TCN structure. Thanks to the PEDOT-Tos layer with excellent NIR light absorption characteristic, the NIR light illumination via a small LEDs array allowed the multiple PEDOT-Tos coated TCN actuators to be photo-thermally heated to a fairly consistent temperature and to simultaneously produce a contractile strain that could be modulated as high as 8.7% with light power. The actuation performance was reversible without any significant hysteresis and highly durable during 3000 cyclic operations via repetitive control of the LEDs. Together with its simple structure and facile fabrication, the light-driven actuator can lead to technical advances in artificial muscles due to its attractive benefits from remote controllability without complex coupled instruments and electromagnetic interference.

## 1. Introduction

Inspired by natural muscles that can deform in response to diverse stimuli for complex locomotion of organism with a lightweight and simple structure, soft actuators mimicking the capability of the natural muscles have been developed by using numerous kinds of functional polymers, such as shape memory polymers, twisted and coiled polymers, dielectric elastomers, hydrogels, liquid crystal polymers, and conducting polymers [1]. Based on their distinctive design and actuation mechanism selectively responding to each external stimulus (heat [2,3], light [4,5,6], solvent [7,8], electric power [9,10], magnetism [11,12], and moisture [13]), the soft actuators have shown their potentials in realization of tensile, contractile, bending, and rotational deformation. Moreover, unlike the conventional hydraulic/pneumatic or electro-mechanical actuators, soft actuators that can possess mechanical resilience and produce continuum deformation even with a simple, flexible and lightweight structure have led to advent of many kinds of soft robots: meshworm or rolling robot (SMA) [14,15], serpentine locomotion robot [16], jamming robot [17], jumping robot [18], grasping robot [19,20], and swimming robot [21,22,23].

Compared to the other soft actuators, twisted and coiled polymer actuators (TCPAs) have recently been given much attention as a strong candidate of artificial muscle, due to their reversible and large-stroke actuation performance lifting a heavy load comparable to that of human muscle. Many fibrous materials such as, polyethylene, nylon, silk, and cotton have been studied for the TCPAs and demonstrated their functionality for the promising applications such as artificial finger, smart clothing, prosthetic limb, and soft robots [24]. The TCPAs produce large-angle rotation or large stroke contractile actuation through radial volume expansion of fiber typically in response to heat energy. Researchers used various approaches to heat the TCPAs in previous studies, including Joule heating, convective heating, and photo-thermal heating. Particularly, photo-thermal heating has been considered as an attractive way among these heating strategies because it allows the actuation of TCPAs to be remotely controllable without complex coupled instruments and electromagnetic interference. A few researchers have reported sodium polyacrylate (PAAS)/graphene oxide (GO) composite and sodium alginate (SA)/GO composite with twisted configuration, which can produce contractile deformation according to volume shrinkage of the twisted fiber when water molecules absorbed in GO are evaporated during near infrared light illumination [25,26]. However, as far as we know, there is only a study of photo-thermally-driven TCPA via light illumination using incandescent lamp [27].

Here, we construct a thin poly(3,4-ethylenedioxythiophene) doped with *p*-toluenesulfonate (PEDOT-Tos), which possesses excellent light absorption characteristic of near-infrared (NIR) light, on the surface of a twisted and coiled nylon-6 fiber (TCN) with a complex three-dimensional structure via a dip-coating and thermal polymerization (DP) process. We report a novel study for fabrication, photo-thermal heating behavior, and light-driven actuation performance of the PEDOT-Tos coated TCN actuator (PT-TCNA), integrating with a small LEDs array.

## 2. Experimental Section

### 2.1. Materials

Nylon-6 monofilament fishing line (diameter: 370 μm) was purchased from Toray Industries, Inc., Tokyo, Japan (Model: High Position Float). CLEVIOS^TM^ CE, which is a commercially available solution including 40% of iron (III) p-toluene sulfonate (Iron-tosylate) in ethanol, was purchased from Heraeus GmbH & Co. Hanau, Germany. 3,4-ethylenedioxythiophene (EDOT, Mw: 142.18 g/mol), pyridine (Mw: 79.10 g/mol), and triblock copolymer of poly(ethylene glycol)-block-poly(propylene glycol)-block-poly(ethylene glycol) (PEG-PPG-PEG, Mn: ~2800) were purchased from Sigma-Aldrich, Darmstadt, Germany.

### 2.2. Structural Forming of a Twisted and Coiled Nylon-6 Fiber

**Figure 1:**
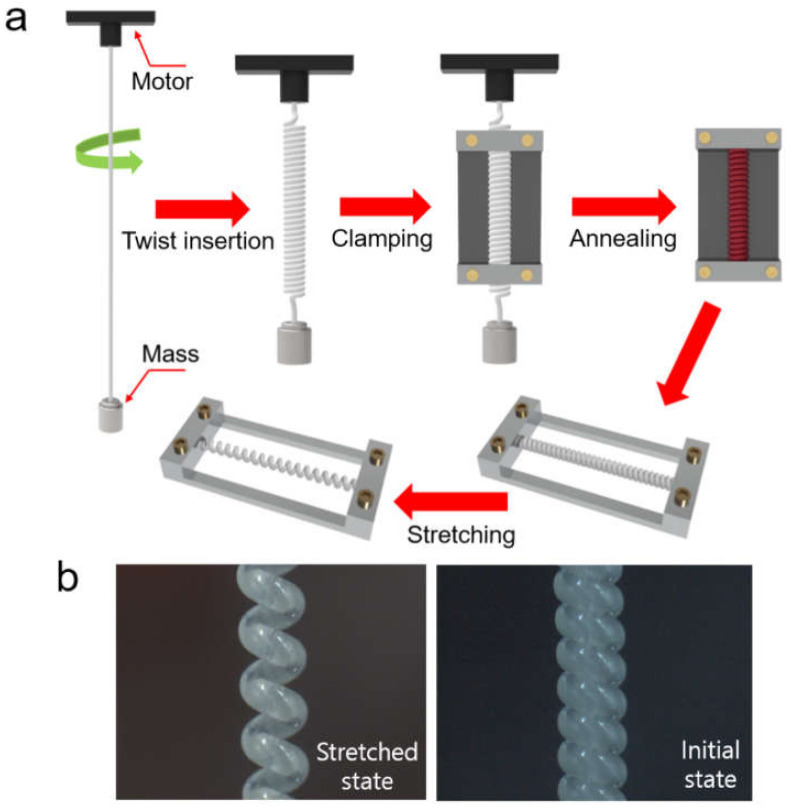
(**a**) An illustrated stepwise fabrication process of TCN structure; (**b**) its optical microscope images taken before and after stretching.

A twisted and coiled structure of a monofilament nylon-6 fiber (length: 1200 mm) was achieved by inserting twist to the fiber with a speed of 700 RPM under a load of 40 MPa. The resulting TCN, which has a length of 300 mm and a diameter of 950 μm, was clamped to a couple of metallic fixtures as maintaining tension, thermally annealed in a vacuum oven at 170 °C for 100 min, and then naturally cooled down to room temperature (25 °C). Figure 1a shows a schematic illustration of a stepwise fabrication process of the TCN. The prepared TCN, which is shown in Figure 1b, was cut into five pieces in a 60-mm length. Each of the pieces were mechanically stretched to 40% and then clamped again to a metallic fixture.

### 2.3. Preparation of PEDOT-Tos Coated TCN

**Figure 2:**
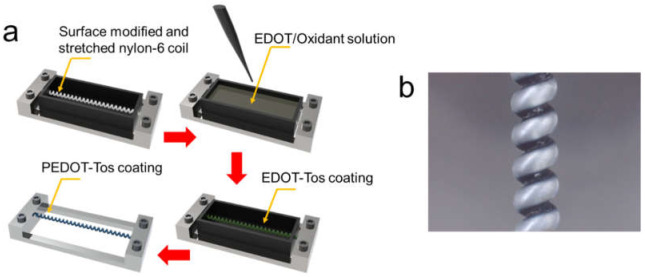
(**a**) A stepwise DP process constructing a thin PEDOT-Tos coating on the surface of the TCN; (**b**) a photograph of the PEDOT-Tos coated TCN taken by optical microscope.

The clamped TCNs were pre-cleaned progressively with acetone and isopropyl alcohol via sonication for 3 min and then dried in vacuum oven. In order to increase surface hydrophilicity, UV-ozone plasma was exposed to the TCN under room temperature for 7 min at a constant vertical distance of 50 mm from a low-pressure mercury vapor discharge lamp (150 W), emitting UV-C light (wavelength: 254 nm) in a YUILUV UV/Ozone system. The surface modified TCN were combined with a rectangular vessel made with a 3D printer. For the dip-coating process, an oxidant solution mixing pyridine (13.5 mg) and PEG-PPG-PEG (200 mg) to CLEVIOS^TM^ CE (1 g) was prepared and then EDOT monomer (44 mg) was added to the oxidant solution. The dark greenish solution was filled into the vessel with the TCN and then it was sucked out of the vessel using a syringe after 3 min. The liquid coating on surface of the TCN was converted into a thin PEDOT-Tos layer (navy-blue color) via thermal polymerization of EDOT with oxidant during drying process in a heating oven at 60 °C for 1 h [28,29]. For eliminating excess oxidants [29], the resulting PEDOT-Tos layer formed on each TCN was washed with ethanol under magnetic stirring for 20 s and dried in a heating oven at 60 °C for 10 min. Figure 2a,b show a schematic illustration of the DP process and an optical microscope image of the PT-TCN fabricated via the DP process, respectively. We modulated thickness of the PEDOT-Tos layer by adjusting the number of repetitions of the DP process.

### 2.4. Characterization

Influence of UV/Ozone plasma treatment on the surface characteristic of the nylon-6 material were evaluated by measuring the water contact angle before and after exposing it to the plasma using a KRUSS DSA 25S drop shape analyzer. For the measurement, in substitution for the nylon-6 fiber, we used a nylon-6 film (GoodFellow, thickness: 350 μm). Surface and cross-sectional morphology of PEDOT-Tos layer covering the nylon-6 fiber as well as TCN was observed through a ZEISS Axio Scope A1 optical microscope and a FEI SIRION 400 scanning electron microscope (SEM).

### 2.5. Performance Test

Either a single PT-TCN or multiple PT-TCNs were clamped to a couple of cylindrical rigid frames at both ends with a 25-mm interval length. Then, a circuit board integrated with a vertical 1 × 5 array of NIR LEDs was inserted into a perforated groove in the upper frame, while keeping a consistent distance (4 mm) between the TCN and LEDs. Finally, the PT-TCNs were vertically-stretched to 16% through a mass loading on the bottom frame. The LEDs had a 5-mm center-to-center pitch and the emitted NIR light covered about 25 mm in length, which is a little shorter than the exposed length of the TCN. Under a NIR light illumination, change in surface temperature on the PT-TCNs was monitored and recorded in 5 frames per second by an Optris PI640 MWIR thermal camera. For the light illumination, we used an individually-controllable 940-nm LEDs (Luminus SST-10-IRD-940 nm B50) with input power of 0–0.5 W per each LED. During on-off cyclic illumination of the NIR light, photo-thermally-induced deformation behavior of the PT-TCNs was evaluated by measuring their contractile strains with a LK-H055 KEYENCE laser displacement sensor.

## 3. Results and Discussion

### 3.1. Constructing PEDOT-Tos Layer on Nylon-6 Material

**Figure 3:**
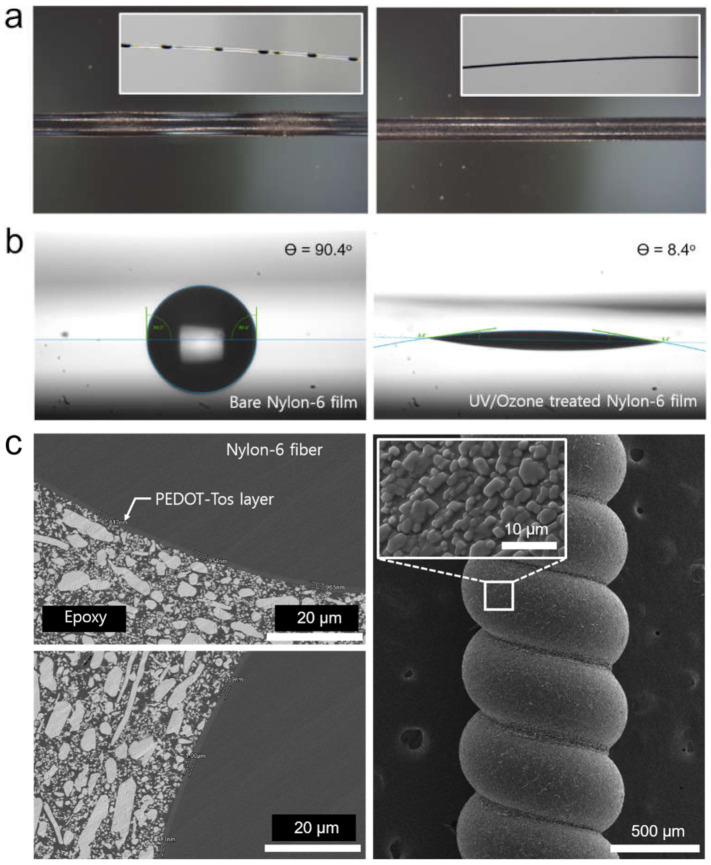
(**a**) Optical microscope images of the PT-TCN TCN prepared before UV/Ozone treatment (left) and after the treatment (right) (insets are low magnification photographs); (**b**) water contact angle at surface of a nylon-6 film before and after UV/Ozone treatment; (**c**) cross-section and surface SEM images of the PT-TCN.

A thin PEDOT-Tos layer was established on the surface of a flat nylon-6 fiber and a three-dimensional TCN via the DP process. In order to investigate the suitability of the DP process for constructing the PEDOT-Tos layer uniformly on the surface of nylon-6 material, we firstly attempted the DP process for a precleaned flat nylon-6 fiber by immersing it into an EDOT/oxidant solution using ethanol as a solvent. As a result, the liquid droplets (dark greenish color) were formed locally on the surface of the nylon-6 fiber, suggesting that the nylon-6 fiber possesses relatively low hydrophilic nature. As shown in Figure 3a (left), the uneven liquid coating caused a PEDOT-Tos layer to be formed bumpily onto the local surface of the nylon-6 fiber after thermal polymerization due to the mismatch in the surface characteristic between the liquid coating and the nylon-6 fiber.

For improving the wettability of the liquid in contact with the nylon-6 fiber surface, UV/Ozone treatment, which is one of effective methods modifying the surface of polymers to have abundant hydrophilic groups (i.e., –OH, –COOH) [30], was performed. Influence of the UV/Ozone treatment on surface characteristic of the nylon-6 fiber was analyzed by measuring water contact angle (WCA) before and after the UV/Ozone treatment. In this study, we used the nylon-6 film in substitution for the nylon-6 fiber due to a difficulty in exploiting the sessile drop method for a single fiber with a small diameter, which is much less than 1 mm. For the test, all specimens were prepared via the precleaning process, which is the same as that of the TCN. As shown in Figure 3b, the bare nylon-6 film exhibited WCA of around 90°, which is considered to be intermediate-wet. However, after UV/Ozone treatment for 7 min, the nylon-6 film became strongly water-wet with WCA as low as 8.4°, indicating that the treatment imparts superhydrophilic nature to the surface of nylon-6 material and it can provide the high affinity of the EDOT-oxidant solution for the surface. Due to benefit from the surface modification, we successfully achieved uniform coating of the PEDOT-Tos on the nylon-6 fiber surface, which is shown in Figure 3a (right).

Based on the study, we attempted dip-coating of the EDOT-oxidant solution on the UV/Ozone treated TCN, instead of the bare one. Particularly, we note that the TCN is axially stretched to 40%, which is much less than its elastic limit, in advance of the UV/Ozone treatment and dip-coating because the mechanical stretching can open a space intentionally among coiled structures of the TCN, which are stuck to each other, allowing the UV/Ozone plasma as well as the liquid to be reached to the veiled surface of the TCN. As a result, the EDOT-oxidant solution was uniformly coated on the surface of the stretched and UV/Ozone treated TCN without any localized droplet formation and it was successfully converted into PEDOT-Tos layer by the DP process. Figure 3c shows cross-section and surface SEM images of the PT-TCN formed via the DP process repeated twice. The SEM observation revealed that the PEDOT-Tos layer (an averaged thickness: 929 nm) was constructed with a small thickness deviation, which is less than 70 nm, on the whole circumference of the TCN fiber. It suggests that the DP process together with UV/Ozone treatment and mechanical stretching can be a facile method to form uniform PEDOT-Tos layer on a complicated three-dimensional structure.

### 3.2. Photo-Thermal Heating Characteristics of the PEDOT-Tos Layer on TCN Structure

**Figure 4:**
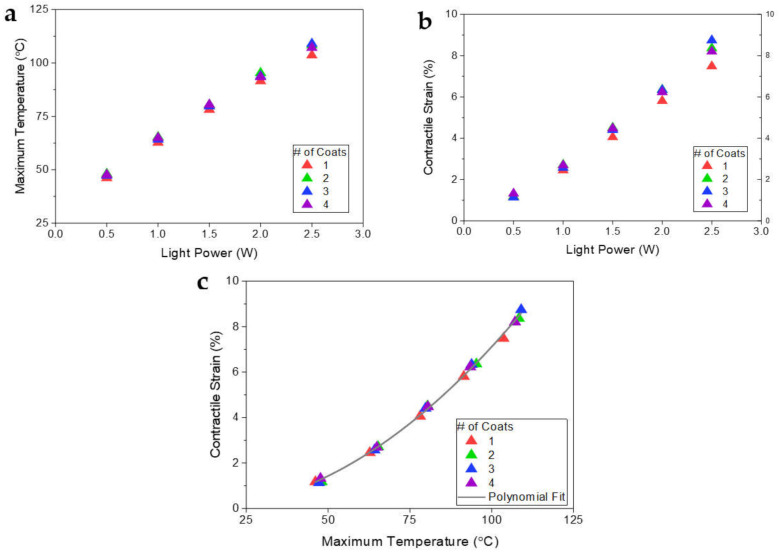
(**a**) Surface temperature profile; (**b**) contractile strain with input light power; (**c**) contractile strain with maximum temperature for PEDOT-Tos coated TCPAs prepared by adopting a different number of the dip-coating process.

Adopting the established DP process with UV/Ozone treatment and mechanical stretching, we prepared the PT-TCNs. Under a consistent NIR light illumination for 25 s, we first evaluated the number of the DP process and light power dependent photo-thermal heating characteristics of the PEDOT-Tos layer formed on the surface of the TCNs. As shown in Figure 4a, the surface temperature increased as high as around 108 °C by increasing the light power from 0.5 to 2.5 W. Under each light power condition, an achievable heating temperature from the PEDOT-Tos layer formed via a single DP process was relatively lower than that formed via multiple DP processes, and their difference in temperature became greater than 4 °C when the light power increased to 2.5 W. On the other hand, the temperature was almost the same once the number of the DP process repeated was equal or more than two. This tendency is strongly inherited from the relationship between thickness of the PEDOT-Tos layer and absorption of the NIR light. When the DP process was repeated twice, the thickness of the PEDOT-Tos layer was close to 1 µm as shown in the previous section, and the absorption could already be high enough for effective photo-thermal heating with only 0.3% of light transmittance at NIR wavelength [31].

Meanwhile, although the difference in achievable temperature between single and multiple DP processes looks quantitatively small, it exerted a substantial influence on the deformation performance of the PT-TCNA. As shown in Figure 4b, when we measured the contractile strain of the PT-TCNAs under a 100 g load by using a laser displacement sensor, all PT-TCNAs produced a photo-thermally-induced contractile strain in proportion to the light power. However, compared to the single DP process, the repetitive DP processes could coherently elicit the strain augmented to as high as 11.8% with a small deviation of 0.87% in most light power conditions except 0.5 W, which is an extremely low power, confining the heating temperature to be below 50 °C. It suggests that the DP process repeated twice is enough to construct the PEDOT-Tos layer on the TCN, securing the superior light absorption capability of the NIR wavelength for the elevating photo-thermal heating temperature further, resulting in amplifying the contractile strain.

The relationship between the maximum temperature and the contractile strain of PT-TCNAs were depicted in Figure 4c with a quadratic fit (*R*^2^ = 0.9996). The slope of strain over temperature increased from 5.07 × 10^−4^/K to 1.04 × 10^−3^/K with the maximum temperature. The strain could be improved effectively via giving more light power, resulting in increasing the maximum temperature until it reaches to an overheat around 120 °C.

### 3.3. Actuation Performance of the PT-TCNA

We fabricated a PT-TCNA by integrating a 1 × 5 NIR LED array into a cylindrical frame holding PT-TCN. The LED array, which is positioned at a consistent distance of 4 mm from the PT-TCN, is capable of illuminating NIR light (wavelength: 940 nm), allowing stepwise control in light power of each LED for photo-thermal heating of the PT-TCNA. By illuminating the LEDs, we first monitored longitudinal temperature distribution onto the single PT-TCNA, which was prestretched to a 29 mm length under a load of 100 g, through an IR thermal camera.

**Figure 5:**
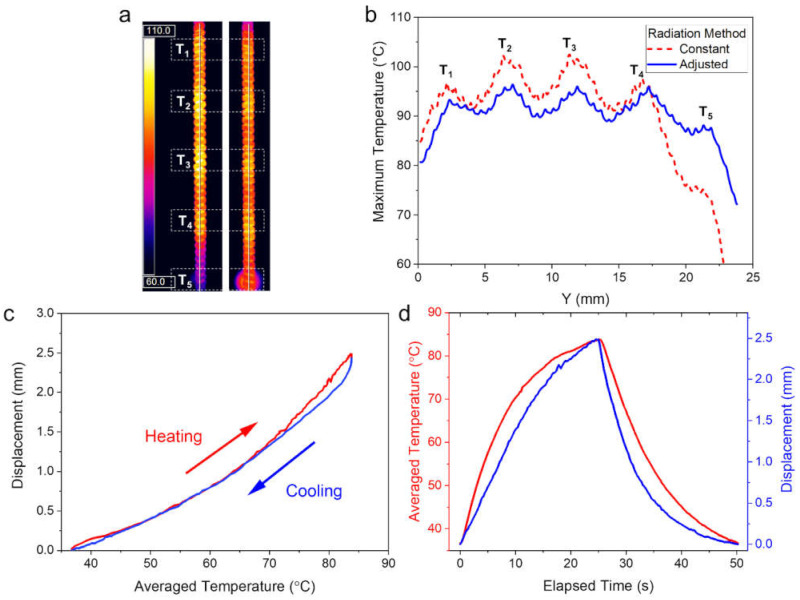
Effect of the spatial light power modulation to equalize vertical temperature distribution of the single PT-TCNA during photo-thermal heating using a 1 × 5 LEDs array: (**a**) Thermal images of a PT-TCNA radiated with a constant light power (left) and the modulated light power (right); (**b**) their vertical temperature distribution over central Y-axis; (**c**) hysteresis of the actuation performance during a heating/cooling cycle; (**d**) temporal change of temperature and displacement during a heating/cooling cycle.

As shown in Figure 5a, when the NIR light was illuminated, the light energy absorbed by the PEDOT-Tos layer was converted into heat energy and it led to photo-thermal heating of the PT-TCNA. However, we found that the temperature distribution onto the PT-TCNA was quite irregular under operation of the LEDs with the same light power. As depicted as the Constant method in Figure 5b, the central area was heated 27 °C higher than the lower end point, which means a 54% higher temperature increment from room temperature, indicating an influence of thermal conduction from neighboring areas heated via other LEDs with an illumination angle. Since the overheated areas could suffer from irreversible distortion in the coiled structure being loosened, resilience of the coiled structure was partially degraded and it caused performance of the PT-TCNA to be unstable even in a small contraction induced from the underheated areas. In parallel, the irregular heating hinders the PT-TCNA from bringing out the highest performance because it compels the upper limit of usable light power to be lowered inevitably for securing stability in operation. Therefore, in order to resolve the irregular heating issue and equalize the temperature distribution on the PT-TCNA, we modulated the input power of each LED to be inverse of the temperature profile. As a result, compared to the case exploiting a constant power, the temperature uniformity was significantly improved while keeping the temperature deviation in ±5 °C over 80% region of the PT-TCNA.

With the improved thermal uniformity, the PT-TCNA exhibited reversible contractile deformation with a small hysteresis during a heating/cooling cycle, as shown in Figure 5c. Following the quadratic relationship between temperature and strain, as shown in Figure 4c, the temperature increased relatively faster than the related contractive actuation when the temperature is below 65 °C, while it showed relatively slower change than the displacement in the high temperature region, about >65 °C. We also represented temporal changes of the averaged surface temperature and displacement of the PT-TCNA during a 50 s cycle in Figure 5d. The time response exhibited that the PT-TCNA could produce deformation with an amplitude profile of the temperature, simultaneously allowing recovery to the initial state. In the time response, the change in temperature slowed down gradually in each of the 25 s long heating and cooling phases, from >4.0 °C/s to <0.5 °C/s. On the contrary, the actuation speed was maintained during the first 10 s, as 0.139 mm/s, and slowed down to 0.055 mm/s at the end of the heating phase. During the cooling phase, the relaxation started with a speed (0.371 mm/s), which is about 2.7 times higher than that of the initial contraction. Then, the speed decreased continuously to 0.010 mm/s with the cooling speed and strain change over temperature.

**Figure 6:**
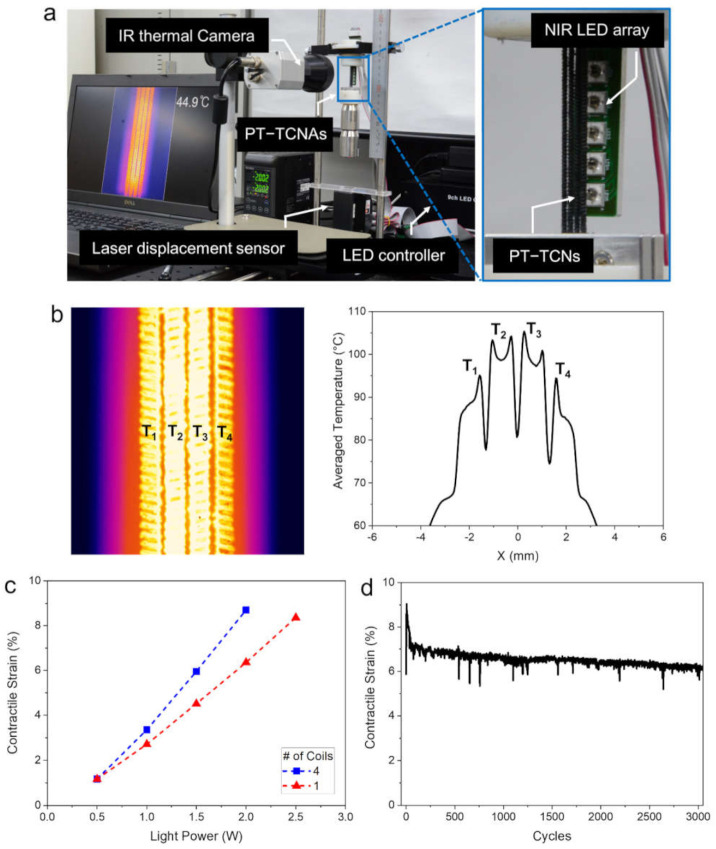
(**a**) A photograph of performance measurement system; thermal image and actuation performance of the multiple PT-TCNAs during light illumination from the LEDs with modulated power; (**b**) thermal image of four PT-TCNs (left) and horizontal temperature profile averaged over vertical axis (right); (**c**) contractile strain profiles with light power; (**d**) change in contractile strain profile during 3000 repetitive actuations.

Meanwhile, the use of LED light as the driving force has an attractive advantage for providing light energy onto a wide area, as it is much larger than the width of a coiled nylon-6 fiber. To exploit this advantage, we additionally investigated light-driven actuation with four PT-TCNAs arranged in parallel using the same LED array as the adopting modulation of their input light powers for securing temperature uniformity. The study was conducted by measuring actuation performance, simultaneously monitoring the temperature distribution on the PT-TCNAs using a test system, which is shown in Figure 6a. Thanks to the benefits from light-driven methodology, allowing a wide area heating, the multiple PT-TCNAs could be heated around 100 °C with a small temperature difference among them of about 10 °C during light illumination via LEDs using an input light power of 2.0 W, as represented in Figure 6b, as a thermal image and a temperature plot averaged vertically. As presented in Figure 6c, the multiple PT-TCNAs can generate a reversible tensile stroke, lifting a load of 400 g with a contractile strain as high as 8.7% at a light power of 2 W, which is 37%, and 5% greater than those of single PT-TCNA at 2.0 W, and 2.5 W under the same 100 g per actuator loading condition, respectively. Video S1 is showing the heating and actuation of the four PT-TCNAs. Furthermore, during 3000 cyclic actuation tests through repetitive On/Off control of the LEDs, the multiple PT-TCNAs generated a stable contractile strain of over 6% of its relaxed length without any significant performance degradation (Figure 6d).

## 4. Conclusions

As a remotely controllable artificial muscle, we developed a novel light-driven PT-TCNA integrated with a NIR LEDs array that can produce tensile stroke in response to light illuminating via LEDs. The PT-TCNA was prepared by constructing uniform PEDOT-Tos layer on a three-dimensional TCN structure via a facile DP process. Thanks to the excellent NIR absorption property of the PEDOT-Tos layer, the PT-TCNA could be photo-thermally heated in response to illumination of the NIR light, allowing stepwise modulation of the heating temperature as high as 110 °C with the light power ranging from 0.5 to 2.5 W. By resolving local overheating issue via modulation of the light power with a gradient, the PT-TCNA could produce reversible and durable contractile strain during repetitive On/Off control of the LEDs. Finally, by exploiting the advantage of the LEDs providing light energy in a wide area, we demonstrated that the multiple PT-TCNAs could produce a reversible tensile stroke, lifting a load of 400 g with a contractile strain as high as 8.7%, 37% greater than that of the single PT-TCNA at the same 2.0 W light power.

## Data Availability

The data presented in this study are available on request from the corresponding author.

## Supplementary Materials

The following is available online at https://www.mdpi.com/article/10.3390/polym14030432/s1, Video S1: Optical and thermal video in contractile operation of PT-TCNAs.

## References

[1] Zou M., Li S., Hu X., Leng X., Wang R., Zhou X., Liu Z. (2021). Progresses in Tensile, Torsional, and Multifunctional Soft Actuators. Adv. Funct. Mater..

[2] Chen Q., Yu X., Pei Z., Yang Y., Wei Y., Ji Y. (2017). Multi-Stimuli Responsive and Multi-Functional Oligoaniline-Modified Vitrimers. Chem. Sci..

[3] Park Y., Gutierrez M.P., Lee L.P. (2016). Reversible Self-Actuated Thermo-Responsive Pore Membrane. Sci. Rep..

[4] Chen L., Weng M., Zhang W., Zhou Z., Zhou Y., Xia D., Li J., Huang Z., Liu C., Fan S. (2016). Transparent Actuators and Robots Based on Single-Layer Superaligned Carbon Nanotube Sheet and Polymer Composites. Nanoscale.

[5] Gao D., Ding W., Nieto-Vesperinas M., Ding X., Rahman M., Zhang T., Lim C., Qiu C.-W. (2017). Optical Manipulation from the Microscale to the Nanoscale: Fundamentals, Advances and Prospects. Light Sci. Appl..

[6] Gelebart A.H., Jan Mulder D., Varga M., Konya A., Vantomme G., Meijer E.W., Selinger R.L.B., Broer D.J. (2017). Making Waves in a Photoactive Polymer Film. Nature.

[7] Zhao Q., Dunlop J.W.C., Qiu X., Huang F., Zhang Z., Heyda J., Dzubiella J., Antonietti M., Yuan J. (2014). An Instant Multi-Responsive Porous Polymer Actuator Driven by Solvent Molecule Sorption. Nat. Commun..

[8] Sun Y.-L., Dong W.-F., Niu L.-G., Jiang T., Liu D.-X., Zhang L., Wang Y.-S., Chen Q.-D., Kim D.-P., Sun H.-B. (2014). Protein-Based Soft Micro-Optics Fabricated by Femtosecond Laser Direct Writing. Light Sci. Appl..

[9] Li Q., Liu C., Lin Y.-H., Liu L., Jiang K., Fan S. (2015). Large-Strain, Multiform Movements from Designable Electrothermal Actuators Based on Large Highly Anisotropic Carbon Nanotube Sheets. ACS Nano.

[10] Amjadi M., Sitti M. (2016). High-Performance Multiresponsive Paper Actuators. ACS Nano.

[11] Fusco S., Sakar M.S., Kennedy S., Peters C., Bottani R., Starsich F., Mao A., Sotiriou G.A., Pané S., Pratsinis S.E. (2014). An Integrated Microrobotic Platform for On-Demand, Targeted Therapeutic Interventions. Adv. Mater..

[12] Hu W., Lum G.Z., Mastrangeli M., Sitti M. (2018). Small-Scale Soft-Bodied Robot with Multimodal Locomotion. Nature.

[13] Han D.-D., Zhang Y.-L., Liu Y., Liu Y.-Q., Jiang H.-B., Han B., Fu X.-Y., Ding H., Xu H.-L., Sun H.-B. (2015). Bioinspired Graphene Actuators Prepared by Unilateral UV Irradiation of Graphene Oxide Papers. Adv. Funct. Mater..

[14] Seok S., Onal C.D., Cho K.-J., Wood R.J., Rus D., Kim S. (2013). Meshworm: A Peristaltic Soft Robot with Antagonistic Nickel Titanium Coil Actuators. IEEE/ASME Trans. Mechatron..

[15] Lin H.-T., Leisk G.G., Trimmer B. (2011). GoQBot: A Caterpillar-Inspired Soft-Bodied Rolling Robot. Bioinspiration Biomim..

[16] Onal C.D., Rus D. (2013). Autonomous Undulatory Serpentine Locomotion Utilizing Body Dynamics of a Fluidic Soft Robot. Bioinspiration Biomim..

[17] Steltz E., Mozeika A., Rodenberg N., Brown E., Jaeger H.M. (2009). JSEL: Jamming Skin Enabled Locomotion. Proceedings of the 2009 IEEE/RSJ International Conference on Intelligent Robots and Systems.

[18] Tolley M.T., Shepherd R.F., Karpelson M., Bartlett N.W., Galloway K.C., Wehner M., Nunes R., Whitesides G.M., Wood R.J. (2014). An Untethered Jumping Soft Robot. Proceedings of the 2014 IEEE/RSJ International Conference on Intelligent Robots and Systems.

[19] Ilievski F., Mazzeo A.D., Shepherd R.F., Chen X., Whitesides G.M. (2011). Soft Robotics for Chemists. Angew. Chem..

[20] Deimel R., Brock O. (2016). A Novel Type of Compliant and Underactuated Robotic Hand for Dexterous Grasping. Int. J. Robot. Res..

[21] Suzumori K., Endo S., Kanda T., Kato N., Suzuki H. (2007). A Bending Pneumatic Rubber Actuator Realizing Soft-Bodied Manta Swimming Robot. Proceedings of the 2007 IEEE International Conference on Robotics and Automation.

[22] Nakabo Y., Mukai T., Ogawa K., Ohnishi N., Asaka K. Biomimetic soft robot using artificial muscle. Proceedings of the IEEE/RSJ International Conference on Intelligent Robots and Systems (IROS2004).

[23] Ogawa K., Nakabo Y., Mukai T., Asaka K., Ohnishi N. A snake-like swimming artificial muscle. Proceedings of the Second Conference on Artificial Muscles.

[24] Leng X., Hu X., Zhao W., An B., Zhou X., Liu Z. (2021). Recent Advances in Twisted-Fiber Artificial Muscles. Adv. Intell. Syst..

[25] Shi Q., Li J., Hou C., Shao Y., Zhang Q., Li Y., Wang H. (2017). A Remote Controllable Fiber-Type near-Infrared Light-Responsive Actuator. Chem. Commun..

[26] Wang W., Xiang C., Sun D., Li M., Yan K., Wang D. (2019). Photothermal and Moisture Actuator Made with Graphene Oxide and Sodium Alginate for Remotely Controllable and Programmable Intelligent Devices. ACS Appl. Mater. Interfaces.

[27] Haines C.S., Lima M.D., Li N., Spinks G.M., Foroughi J., Madden J.D.W., Kim S.H., Fang S., de Andrade M.J., Göktepe F. (2014). Artificial Muscles from Fishing Line and Sewing Thread. Science.

[28] Lim J.A., Park S.H., Baek J.H., Ko Y.D., Lee H.S., Cho K., Lee J.Y., Lee D.R., Cho J.H. (2009). Selectively Patterned Highly Conductive Poly(3,4-Ethylenedioxythiophene)-Tosylate Electrodes for High Performance Organic Field-Effect Transistors. Appl. Phys. Lett..

[29] Lim H., Park T., Na J., Park C., Kim B., Kim E. (2017). Construction of a Photothermal Venus Flytrap from Conductive Polymer Bimorphs. NPG Asia Mater..

[30] Lee M., Lee M.S., Wakida T., Tokuyama T., Inoue G., Ishida S., Itazu T., Miyaji Y. (2006). Chemical Modification of Nylon 6 and Polyester Fabrics by Ozone-Gas Treatment. J. Appl. Polym. Sci..

[31] Hwang I., Kim H.J., Mun S., Yun S., Kang T.J. (2021). A Light-Driven Vibrotactile Actuator with a Polymer Bimorph Film for Localized Haptic Rendering. ACS Appl. Mater. Interfaces.

